# Overcoming language barriers in community-based research with refugee and migrant populations: options for using bilingual workers

**DOI:** 10.1186/1472-698X-14-11

**Published:** 2014-04-12

**Authors:** Susan K Lee, Cheryl R Sulaiman-Hill, Sandra C Thompson

**Affiliations:** 1 Community Development Services Manager, Women’s Health and Family Services, Perth, Western Australia; 2 Research Fellow, Combined Universities Centre for Rural Health, University of Western Australia, Perth, Western Australia; 3 Winthrop Professor, Chair of Rural Health, University of Western Australia and Director, Combined Universities Centre for Rural Health, Geraldton, Western Australia

**Keywords:** Bilingual workers, Cross cultural research, Migrants, Refugees, Communication, Interpreting

## Abstract

**Background:**

Although the challenges of working with culturally and linguistically diverse groups can lead to the exclusion of some communities from research studies, cost effective strategies to encourage access and promote cross-cultural linkages between researchers and ethnic minority participants are essential to ensure their views are heard and their health needs identified. Using bilingual research assistants is one means to achieve this. In a study exploring alcohol and other drug service use by migrant women in Western Australia, bilingual workers were used to assist with participant recruitment and administration of a survey to 268 women who spoke more than 40 different languages.

**Discussion:**

Professional interpreters, bilingual students, bilingual overseas-trained health professionals and community sector bilingual workers were used throughout the research project. For the initial qualitative phase, professional interpreters were used to conduct interviews and focus group sessions, however scheduling conflicts, inflexibility, their inability to help with recruitment and the expense prompted exploration of alternative options for interview interpreting in the quantitative component of the study. Bilingual mature-age students on work placement and overseas-trained health professionals provided good entry into their different community networks and successfully recruited and interviewed participants, often in languages with limited interpreter access. Although both groups required training and supervision, overseas-trained health professionals often had existing research skills, as well as understanding of key issues such as confidentiality and referral processes. Strategies to minimise social desirability bias and the need to set boundaries were discussed during regular debriefing sessions. Having a number of workers recruiting participants also helped minimise the potential for selection bias. The practical and educational experience gained by the bilingual workers was regarded as capacity building and a potentially valuable community resource for future health research projects.

**Summary:**

The use of bilingual workers was key to the feasibility and success of the project. The most successful outcomes occurred with students and overseas-trained health professionals who had good community networks for recruitment and the required linguistic skills. By describing the advantages and disadvantages encountered when working with bilingual workers, we offer practical insights to assist other researchers working with linguistically diverse groups.

## Background

In cross cultural research some topics may be considered more sensitive by some cultural groups than others. One such topic is alcohol and other drug use. Topics are generally considered sensitive when they are regarded as private, involve stigmatised behaviours or evoke strong emotional feelings [1]. Participants in interviews and focus groups may dislike or find it difficult to talk about such a topic, perhaps because the subject is not normally publicly discussed and participants may fear that their experiences, views or beliefs are not normal. Researchers need to be tactful about introducing research on sensitive issues to potential participants, but at the same time should not attempt to hide the nature of the research [2,3]. For research involving sensitive issues, participants may lack the vocabulary (even in their own language) to discuss such issues and they may have never talked about such things prior to participating in the research. This means they may have difficulty articulating their experiences [1]. Bilingual interviewers can assist in helping gather information on sensitive topics, however, these workers need to be suitably chosen as participants may fear disapproval or reprisal after disclosing activities that do not follow a group’s expectations or social norms for example, alcohol use by a participant whose religion prohibits alcohol consumption [1,2]. Thus, the choice of suitable bilingual workers can impact on the ultimate success or failure of a research project. This paper looks at the impact of the use of bilingual interviewers in the latter two phases of a research project with migrant and refugee women in Western Australia examining what can be a sensitive issue, alcohol and drug use by newly arrived women. By describing the advantages and disadvantages of working with the different bilingual interviewers on this project, we offer practical considerations for other researchers working with linguistically diverse groups.

### Interpreters, translators and bilingual/bicultural workers

Bilingual interviewers are often referred to as interpreters, translators or bilingual/bicultural workers. Although these terms are often used interchangeably in the literature, in the Western Australian context they have specific meaning.

#### Interpreters

The WA Health Services Language Policy 2011 [4] aims to ‘facilitate effective communication between health service providers and people needing language assistance.’ It addresses the minimum standards required in the State Language Services Policy 2008 and stipulates when language services ‘must’, ‘should’ or ‘may’ be used within the health context, based on legislative standards. The roles of interpreters and translators are also defined, and minimum standards outlined. Thus, an interpreter is a person who conveys a message or statement verbally or by using sign language between two or more parties using English and another language. Interpreters, who must meet minimum standards of proficiency in both English and a community language, are also trained in skills such as memory retention, turn taking, appropriate terminology and the degree of formality to be used in a given interpreting situation [5]. In Australia, interpreters are accredited nationally and must abide by a code of ethics that includes confidentiality, impartiality and accuracy [5]. In a health care setting, the Language Policy [4] stipulates the type of circumstances where an accredited interpreter should be used, and can include situations where, in a health professional’s assessment, a client has inadequate understanding of critical information to give informed consent. Interpreters are not permitted to complete forms or questionnaires. In these situations a health or social service worker must verbally ask clients each question on the form, the interpreter then interprets the question and the client’s response, which is then recorded. Thus, the health professional’s role is to conduct the interview and then debrief the interpreter at the end. Health researchers using interpreters in Western Australia are expected to follow the same guidelines. As noted elsewhere [6], good interpreters do not offer contextual or cultural meanings of words and phrases unless asked. In Western Australia, this usually would be provided to the service provider in the debriefing session after the participant(s) had left.

#### Translators

In contrast, as defined in the WA Health Services’ Language Policy [4] a translator is a person who makes a written transfer of a message or statement between English and another language. Like interpreters, effective translations must be done with accuracy and impartiality. Translators are trained and accredited through a similar process as interpreters but work only on written documents. In a research setting for example, a translator may translate a questionnaire from one language to another, but like interpreters, they are not able to administer the questionnaire to participants.

Translating questionnaires can add significant monetary costs and time considerations to health research projects, while the translations themselves may also be problematic. Concerns around the appropriate level of language can occur if it is too academic so that the meaning is not clear to the majority of its intended audience [7]. Similarly, colloquial expressions used in one language may not have an exact equivalency in another. The translator, in an attempt to convey the significance of a phrase, could also unintentionally change the meaning [8]. Different versions of a language may also exist in different countries, often with country-specific expressions, thus a translation may not necessarily be understood by all speakers of a language [9]. Ideally, a translated document should be back translated to ensure equivalency and may also be given to community members for feedback on clarity and meaning for non-health professionals [10]. However, because this can result in disagreement between translators, or between translators and community members on the choice of wording, a resolution process, as well as time, is needed to clarify these issues.

#### Bilingual/bicultural workers

In the context of health-related research, bilingual workers are individuals who can communicate in English and another language (or a dialect of English) appropriate to their role as a research assistant for a specific project [5]. They often operate independently of the principal researcher, to conduct interviews or gather other types of information.

Compared with interpreters, there is no nationally accredited training, assessment, and registration process for bilingual/bicultural workers in Australia [5], so their abilities can vary. Although they do not have a common code of ethics, many may still be governed by the relevant professional standards and legislative requirements of their profession, such as social workers or psychologists [5]. In general, bilingual workers cannot be used to communicate information where the potential for misunderstanding puts the employer, client or a worker at high risk, for example, an interpreter rather than a bilingual/bicultural worker must communicate information that is legally binding or when obtaining, communicating or receiving information to make informed decisions [5].

One concern with using bilingual workers is that assessment of language proficiency in languages other than English can be difficult. In Australia, because there is no formal, national registration process [5], one common and accepted method of assessing a person’s proficiency in another language is to use the level of educational qualifications attained overseas, or base it on their overseas work experience [5]. If someone has been educated in a community language, there is an assumption that they will be literate in that language and will have acquired a more formal vocabulary. However, this method gives potential employers of bilingual/bicultural workers little insight into how proficient they will be discussing health or legal issues [5]. If potential workers have vernacular language skills only, without any formal education in that language, this could prove problematic for health research purposes if they lack the health vocabulary required or use less formal language which could have derogatory undertones [11].

Bilingual workers can provide general cultural information as well as information about the communities of interest [5]. They can also help to clarify cultural concepts, the meaning of words and phrases, and provide context for the information the research project is gathering. Some languages, such as French for example, may be spoken in many regions of the world, but cultural factors can vary and knowledge of a particular culture could be important to some research projects. Hence cultural knowledge, not just language proficiency [5] is provided by bilingual/bicultural workers.

Working with interpreters and/or bilingual/bicultural workers to gain information from participants, either through interviews, focus groups or by administering a questionnaire allows people to participate in research that might otherwise be excluded due to a language barrier. However, whatever type of bilingual worker that is used, researchers need to acknowledge that there is often no right way to interpret and/or translate concepts across cultures. Gaining equivalence of meaning across languages is difficult especially when collecting data in one language and reporting it in another [12,13]. People’s lives and experiences influence the way in which they translate and interpret the questions they ask and the responses they are given. In addition, some health concepts or words many not exist in a language making it difficult to convey the meaning from one language to another [14]. Thus, the bilingual worker or interpreter is not neutral, but rather a participant in the research [13] and this needs to be taken into consideration during analysis. Researchers need to consider how workers will understand the meaning of the questions and the answers as part of their training and follow-up sessions. This can offer an insight into possible different perspectives on the research findings [12]. In this way, workers can be more involved in the research process than just collecting the data. They can provide “inside knowledge” that is useful in explaining issues that might not be apparent to an “outsider”. Such inside knowledge balances the outsider perspective of the researcher, allowing data to be viewed from different perspectives. In this way, bilingual workers complement the research process and the result is research with greater depth. The literature has highlighted factors that researchers need to be aware of when working with bilingual workers on research projects and these are the basis of better practice elements in the area of cross cultural health research. These elements are outlined in Table 1.

**Table 1:** Better practice in working with bilingual worker in research

A bilingual worker’s competency in the language of interest to the research project and the researcher’s language e.g. English is assessed in some way. Researchers are aware that a community language acquired in a host country by a migrant can be different than language acquired in a country of origin.	Shimpuki & Norr 2012 [15]
	Hanna et al. 2008 [16]
	Temple 2006 [17]
	Centre for Ethnicity and Health 2008 [5]
Consideration is given to the characteristics of a bilingual worker compared to the participants and how these may impact the study. Some characteristics may need to be matched e.g. age, gender, cultural background.	Shimpuki & Norr 2012 [15]
	Fryer et al. 2011 [18]
	Walin & Ahlstrom 2006 [19]
	Baird 2011 [20]
	Temple 2006 [17]
	Kirkpatrick & Van Teijlingen 2009 [21]
	Berman & Tyyska 2011 [22]
Researchers have considered other factors that are likely to increase the quality of the data collected; for example, data collection is carried out in a safe environment or there is cross checking of data between bilingual workers. Bilingual workers are aware of how these factors can influence the quality of the information they collect.	Walin & Ahlstrom 2006 [19]
	Berman & Tyyska 2011 [22]
Consideration is given as to whether it is important to recruit bilingual workers who have previous experience conducting research and/ or working in the health area related to the research project.	Shimpuki & Norr 2012 [15]
	Squires 2008 [6]
	Baird 2011 [20]
	Centre for Ethnicity and Health 2008 [5]
The researchers provide training, or at least a briefing, about the research project to the worker as well as discussing the bilingual worker’s role and what is expected e.g. timelines.	Shimpuki & Norr 2012 [15]
	Hanna et al. 2008; [16]
	Walin & Ahlstrom 2006 [19]
	Baird 2011 [20]
	Centre for Ethnicity and Health 2008 [5]
Consideration is given to whether a bilingual worker may know the participants and if social desirability bias may influence the quality of the data collected. Researchers are aware that recruitment of participants may be extremely difficult without a worker who knows potential participants.	Walin & Ahlstrom 2006 [19]
	Hanna et al. 2006 [7]
Researchers consider how they will include participants who are illiterate in their own language. The researchers and the bilingual worker discuss this issue and how it will be handled.	Hanna et al. 2008 [16]
There are regular meetings between researchers and bilingual workers to discuss the research process and progress. This also allows for the early identification of any additional training requirements or potential problems.	Shimpuki & Norr 2012 [15]
	Hanna et al. 2008 [16]
	Berman & Tyyska 2011 [22]
The research is collaboration between the researchers and bilingual workers. The bilingual worker’s opinions are valued with regards to the development of focus group questions, questions in survey instruments, and other documents related to the study, such as consent forms. The worker’s feedback about the recruitment process, interviews, and data collected is incorporated into the findings of the study.	Shimpuki & Norr 2012 [15]
	Hanna et al. 2008 [16]
	Baird 2011 [20]
	Hanna et al. 2006 [7]
	Kirkpatrick & Van Teijlingen 2009 [21]
The researcher will not place a bilingual worker at risk. This risk includes damage to their reputation or to negative community comment.	Berman & Tyyska 2011 [22]
The number of bilingual workers used is small to increase the dependability and credibility of the data	Walin & Ahlstrom 2006 [19]

As health researchers we have worked with many different interpreters and bilingual/bicultural workers across a number of projects. In this paper we reflect on how successful or otherwise we were in incorporating better practice elements in working with bilingual interviewers in a study that explored the alcohol and drug concerns of migrant women.

#### Perth migrant Women’s AOD project

Women’s Health and Family Services (WHFS) is a non-government organization providing a range of health, counselling, information and outreach services to women in Western Australia. WHFS works with women from over sixty different nationalities, including both refugee and migrant women. Although new arrivals accessed a wide range of WHFS programs, it was noted that ethnic women were under represented as clients of the alcohol and other drug (AOD) services offered by the organization in Perth. To explore the reasons for this, a research project was undertaken with recently arrived women to examine the prevalence of alcohol and drug use among new arrivals, to identify some of the barriers to AOD services that newly arrived women encountered and to explore the types of services and programs that women wanted. The study received ethics approval from the Human Research Ethics Committee at Curtin University.

The project comprised four phases: an initial community consultation process and establishment of a reference group of culturally and linguistically diverse (CaLD) women; interviews with migrant and refugee social service and health providers; focus group and interview sessions with 26 migrant and refugee women; and a questionnaire-based survey of 268 women. Information on the reference group and findings on the types of services and programs that women wanted are reported elsewhere [23,24]. During the latter two phases of the project, a combination of professional interpreters and bilingual workers were used. The advantages and disadvantages of working with each group have been noted from a research perspective.

### Focus groups and interviews with community women

The sample for both focus groups and the questionnaire survey was limited to women 18 years or older who were permanent residents, or in the process of obtaining residency in Australia, and who had been in Australia 5 years or less. All groups and interviews took place in the Perth metropolitan area. Participants were recruited through the networks of Women’s Health and Family Services (WHFS) using a snowball sampling method. The snowball sample was purposive and used multiple starting points to help ensure participants had a range of opinions and experiences and to limit the bias inherent in snowball sampling [25]. These starting points included WHFS programs, other service providers who provided access to women’s groups and through the networks of the bilingual workers assisting the project. The views and experiences of 26 newly arrived women from a variety of culturally and linguistic backgrounds were collected through one-on-one interviews (n = 6) and through focus groups (n = 5).

One consideration in formulating the project was that the methods used needed to be fair and equitable to all. If one group of new arrivals appeared to receive more attention, information, and/or services it could have had serious negative implications, not only on the viability of the research, but also potentially damaging a worker’s or an organisation’s credibility within the community [26]. For this reason the project did not focus on a specific ethnic group, but rather looked at newly arrived women overall. Although having an ethnically homogenous sample would have been easier for data collection and analysis, it would not have provided an accurate reflection of the women accessing WHFS and their issues. The sample also needed to reflect newly arrived women in the WHFS catchment area. Thus, the research aimed to reflect the population accessing or potentially accessing WHFS programs in order to obtain more meaningful research findings and hence the need to recruit newly arrived women from different source countries, different migration categories and with different life experiences [27].

The use of focus groups and in-depth interviews provided flexibility to accommodate women’s schedules and adaptability in the study design. This was necessary as it was not known whether women would openly discuss issues that might be considered shameful, private or stigmatising in a group setting. Some literature had suggested that this could be the case with this area of research [1,2] and that having a more flexible study design would be helpful. Child care was made available to participants for interviews, if required. Participants were given the choice of participating in the focus groups and interviews in English or through an interpreter from the Translating and Interpreting Service (TIS). Further details about the interviews and focus groups and use of interpreters are provided in Table 2 and shows recruitment from naturally occurring groups that met for other purposes, such as sewing or English classes. Another source was ‘constructed groups’ which refers to groups that only came together for the purposes of the project; these usually yielded more information. Although Perth is a capital city of over one million people, its geographical isolation means that in small minority groups people tend to know each other, and this frequently occurred in the constructed groups.

**Table 2:** Community focus groups

**Focus Group or Interview**	**Agreed to be recorded**	**Women were from**	**Language of Focus group or Interview**
Focus Group: Natural Occurring	Yes	Iraq	Arabic with Interpreter
Focus Group: Natural Occurring	Yes	India	English
		Nigeria	
Focus Group: Constructed	Yes	Macedonia	English
		Liberia	
		Sudan	
		Congo	
		Myanmar (Burma)	
Focus group: Constructed	Yes	Russia	English
		Ukraine	
		Kazakhstan	
		Thailand	
		Afghanistan	
Focus Group: Natural Occurring	Yes	Indonesia	English
		Iran	
		Thailand	
		China	
Interview	Yes	Indonesia	Indonesian with Interpreter
Interview	Yes	Congo	English and French No Interpreter (Both the participant and the interviewer spoke English and French)
Interview	No	Burundi	English
Interview	Yes	Sudan	Arabic with Interpreter
Interview	Yes	Macedonia	English
Interview	Yes	Sudan	Arabic with Interpreter

### Questionnaire survey

The information gathered during these interviews and discussions informed the design of a questionnaire which aimed to clarify the extent to which the issues, attitudes and perceptions explored during the interviews and focus groups were applicable to a broader cross section of newly arrived women. Some questions were taken or modified from questionnaires used elsewhere in Australia such as the National Drug Strategy Household Survey [28] and the WA Health and Wellbeing Surveillance System survey [29] to allow comparison with relevant Australian population ranges and distributions. The questionnaire also included questions exploring specific issues identified from the focus groups and interviews and was constructed to be sensitive to the meanings and interpretations that respondents might place on questions and to avoid responses that might otherwise have been ambiguous or misunderstood [30]. A mixture of open and closed questions was used.

As part of the development process community leaders, service providers and a steering committee of professionals with relevant expertise, were asked to comment on the wording and content of the questionnaire. Piloting of the questionnaire with community women from several CaLD communities occurred following this. Thirteen individuals were interviewed who came from Botswana, Kenya, Ethiopia, the United Kingdom, New Zealand, Indonesia, South Africa, and Germany. Two women interviewed for the pilot were from Australia with overseas born parents. They were asked to comment on the wording of the questions, length of the questionnaire, potentially embarrassing or sensitive questions, the usefulness of the questions, and any other comments on the issues discussed. As a result of these consultations, many of the questions were reworded and simplified, for example, service providers suggested it would be better to ask a woman’s age, rather than the year they were born, as some women did not know the year of their birth but had a rough idea of their age.

The piloting and the final survey was administered by bilingual workers and interpreters, using face-to-face interviews. As a result of the pilot, the questionnaire was reduced in length. Many comments about wording showed the original questions did not have the same meaning to women from different cultural and language backgrounds. For example, many women in the pilot found the words mental health and mental wellbeing difficult to understand. These words were almost all dropped from the questionnaire and replaced by terms such as sad, anxious, worried, and stressed which have been shown in other studies to carry the same meaning in other languages [31].

The pilot also helped to confirm the decision that questionnaires should be completed by interview. There were several reasons for using an interview process. Firstly, many new humanitarian entrant arrivals at the time of the study were illiterate in their own language and/or had good English oral language skills but limited English literacy. Having the questionnaire completed by interview allowed their issues and concerns to be included in the data. Filling in forms could also be threatening for some participants due to past experiences where written information may have led to reprisals or persecution. Using interviewers to administer the questionnaire verbally had, in other researchers’ experience, helped overcome these issues [2,32]. Feedback also revealed that the questionnaire was quite long. Other research suggested using face to face interviews to obtain a higher rate of completed questionnaires, especially for longer surveys [33]. The development and piloting of the questionnaire was time consuming, but resulted in a questionnaire that was well understood by women from different cultural and socio-economic backgrounds and it also increased the likelihood that the results of the main survey would be credible [6].

The final survey was completed by 268 newly arrived women. The age, time in Australia, visa categories, and education levels of the 268 women survey respondents is described in Table 3. Women were born in 50 different countries and spoke 48 different primary languages. Just under half (43%) were humanitarian arrivals (refugees).

**Table 3:** Profile of respondents*

	**Migrant women (n=268)**	
	**Number**	**Percentage**
**Time in Australia**		
6 to 12 months	54	20.4
1 to 2 years	73	27.5
2 to 3 years	63	23.8
3 to 4 years	40	15.1
4 to 5 years	35	13.2
**Total**	**265**	
**Age group**		
18 to 29 years	113	44.8
30 to 39 years	84	33.3
40 to 49 years	43	17.1
50 years or more	12	4.8
**Total**	**252**	
**Region of origin**		
Africa	102	38.0
Asia	80	30.0
Middle East	26	9.7
Russia and former USSR	26	9.7
South and Central America	17	6.3
Europe	15	5.6
Other	2	0.7
**Total**	**268**	
**Visa category**		
Humanitarian	109	43.3
Student	38	15.1
Family/partner/fiancé	76	30.2
Business/skilled migrant	20	7.9
Other	9	3.5
**Total**	**252**	
**Highest level of education completed**		
Some Primary school	18	7.1
Finished Primary school	12	4.7
Year 10 or below	26	10.2
Year 12 or below	71	27.8
Trade/TAFE/Apprenticeship	37	14.5
Bachelor Degree	67	26.3
Post Graduate Degree	24	9.4
**Total**	**255**	

*Numbers do not total to 268 because of missing data.

There were 19 bilingual/bicultural workers involved with piloting and administering the questionnaire. The majority of interviews (82.5%) took place in English or a mixture of English and a community language. Thirty interviews were conducted exclusively in a community language using bilingual/bicultural workers and 10 interviews were conducted using an interpreter.

## Discussion

The migrant women’s AOD study employed both professional interpreters and a range of bilingual workers from different backgrounds to assist with various aspects of the project. The profiles, advantages and disadvantages of each group are outlined below.

### Professional interpreters

Eleven female interpreters were employed for the study, in the qualitative phase where they assisted with the interviews and focus group sessions (see Table 2), and also during the quantitative phase of the project.

Quality assurance was the main advantage in working with professional interpreters; to provide confidence that what was being asked was interpreted into the participant’s language as accurately as possible. This was particularly helpful during the qualitative phase of the project when broad themes were being explored and questions piloted for the upcoming survey. In some cases during the quantitative phase of the project, interpreters provided the only opportunity for some women to participate when there was no match between the potential participant and any of the bilingual/bicultural workers on the project.

The main disadvantage encountered with busy professional interpreters was inflexibility with scheduling appointments and their inability to actively participate in the research due to the contractual obligations and legislative framework under which they are employed. In some cases it proved difficult to locate a specific language interpreter at a time convenient to the participant, or the interpreter would be delayed by another appointment. At other times, participants forgot or needed to reschedule the appointment due to family commitments which necessitated renegotiating the interview time for everyone involved, and potentially incurred cancellation fees. In some new and emerging language groups accredited interpreters did not exist and the women who spoke these languages would therefore be excluded from the research if alternative options for interpreting were not available. As these were often the very women the research was trying to reach, any requirement to use only professional interpreters would have had a negative impact on the project findings overall.

A number of unknown impacts of using interpreters in a project such as this one were revealed that require further research. In general, interpreters were older women, well established and resident in Australia longer than the participants they interpreted for. It is unknown whether this impacted on the information relayed. There may have been a greater potential for social desirability bias if younger women did not want to appear ignorant or to be breaking cultural norms to older, respected women. On the other hand, because the interpreters did not necessarily belong to the same social community as the participants they may have been perceived as more neutral and encouraging greater freedom for participants to say what they felt [22].

Scheduling conflicts, inflexibility and costs encouraged us to explore alternative options for interpreting. While professional interpreters fulfil the technical requirements, for some research projects it may not be logistically feasible to use them on an ongoing basis. Apart from the financial constraints facing research projects with minority groups, accredited interpreters are not available for participant recruitment, in-depth conversations to explore cultural meanings, or to provide background information on events in the local community that could impact on participation or responses; this is the role of a bilingual/ bicultural worker.

### Bilingual workers

Nineteen bilingual/bicultural workers were recruited through the networks of WHFS as, unlike interpreters, there is no agency in Western Australia that brokers such groups. All workers recruited had either a health or social service background as they needed to feel comfortable discussing issues around alcohol and other drug use which is a highly sensitive and stigmatised topic for some cultural groups. This, combined with the training they received, was essential to help reduce the chance of interviewers skipping or skirting around certain questions that would be normally uncomfortable to discuss [1,9,27,34].

We tried to ensure most workers had a similar background to the participants, culturally and linguistically, but also in the time they had been in Australia and their level of settlement. Other researchers have suggested that sharing a similar background helps participants build better rapport with the interviewers and increases the likelihood of discussing sensitive topics more openly [22].

All bilingual workers attended a training session which was conducted by the principal researcher. Training covered administration of the survey, issues of confidentiality, asking but not coercing participants, aspects of personal safety when administering the questionnaire, how to respond to participants who became distressed and the criteria for referral to Women’s Health Services for further assistance if required. In addition, each question in the survey was reviewed as to its meaning, and why it was important to ask the question. After the training session, workers were given a small number of surveys, usually five, to complete with women from their own communities, and they were then debriefed to discuss any issues arising during the interview process and to answer outstanding questions. The debriefing process also provided feedback to help better understand the meaning of responses [18]. The bilingual workers were then given the option of completing further surveys with other women from their community.

The bilingual/bicultural workers fell into three broad categories in terms of their background. The advantages and disadvantages of using bilingual/bicultural workers of different backgrounds were noted in the research project and are explored here.

### Students

The first group were students on placement at WHFS while completing Certificate or Diploma level courses in Community Services at local Technical and Further Education (TAFE) centres. These were all mature age students, who had primarily come to Australia as humanitarian entrants. Their community language competency was ascertained by the fact that all of the students had completed high school or tertiary courses in their own countries of origin and often had worked for many years in their country of origin or in refugee camps. Their English language competency was ascertained to be of a sufficient level as they were completing post-secondary qualifications in Perth.

Most students involved in the research project had very good community networks, which provided access to women who would not normally be involved in a project such as this. The students felt they were doing something useful both for themselves and their community, and that the experience of interviewing was of practical utility for their chosen field of study. The student interviewers commented that they enjoyed the survey experience overall, and most were able to easily complete the required number of questionnaires, often more. In many cases the interview process helped to highlight issues that had been covered in their course work, such as confidentiality, the role of the worker, maintaining boundaries, dealing with ethical issues, and self-care. Students were asked to reflect on the positive and negative aspects of being involved in this type of research project during regular group debriefing sessions when they also had opportunities to individually discuss their experiences of the interview process [15]. Discussions covered a wide range of issues such as difficulties in recruitment of potential participants, ways of avoiding social desirability bias, and some of the challenges of working with members of their own ethnic community. Several students described community expectations for them to “fix the system” in which new arrivals and their families were struggling, with community women expecting an almost immediate response to the issues they raised in a much shorter time frame than possible. Students also had to deal with criticism that the survey was largely about alcohol and drug issues, especially as consumption of drugs or alcohol is frowned upon by many new arrival groups.

From an organisational perspective, the student placements were time consuming to supervise and organise, especially as they needed to have multiple tasks to occupy them between their research-based commitments. As an agency, WHFS facilitates many student placements each semester, especially for those who have English as a second language who often need extra support and encouragement to complete their required placement hours. Increasing the opportunities for students to gain exposure and experience in dealing with alcohol and drug issues was seen as assisting CaLD women to gain knowledge and experiences that could be used both informally and formally within their own communities. However, not all students had good community networks, especially those who had recently arrived in Australia. Another disadvantage, as other researchers have found [35], is that students often leave a project at the end of a semester or year to return home, gain employment or simply continue on with the next stage of their course. Thus, there is the need to recruit more students if a project runs over several semesters. This was also our experience in using students.

### Overseas trained health professionals

Another broad category of bilingual/bicultural workers was overseas trained health professionals, including doctors, nurses and psychologists who, as new permanent residents of Australia, were in the process of requalifying to work in their chosen profession in Western Australia. The majority of these overseas trained health and social welfare professionals were either humanitarian entrants or spousal visa holders and were facing numerous barriers to re-qualification. Their community language competency was ascertained by the fact that all of these professionals had studied at tertiary level in their own countries and had many years of professional experience in their country of origin prior to moving to Australia. Their English language competency was ascertained to be of a sufficient level as they were all studying for a higher level English exam (IELTS) in order to undertake the process to register as a health or social welfare professional in Western Australia.

The advantage of this group was their excellent grasp of health related issues as well as concepts such as confidentiality which meant that the focus of their training was primarily on questionnaire administration. In addition, these health professionals had better referral skills, and could handle potential problematic situations better than the student group who had only limited client-related experience. Given their professional backgrounds, this group needed far less supervision and support than the student bilingual workers. Because some of the women were also enrolled in post graduate studies, participating in the study proved a useful means for them to gain practical research experience for their own upcoming research project.

The women recruited through the professional network were employed casually on an hourly rate, and for many this was their first paid job in Australia. From an administrative perspective, the main disadvantage using this group was the additional time required to orientate them to the Australian employment system, which in many cases, was considerably more time consuming than survey administration and the associated follow-up supervision related to the actual project.

### Community sector bilingual workers

The last broad category of bilingual/bicultural workers was women who were either current or previous employees of social service agencies. There were three bilingual workers in this category. All had good community networks and had assisted with previous research projects conducted by universities or social service agencies. Their community language competency was ascertained by the fact that these workers were either employed currently or in the past as bilingual workers at other agencies or researchers, while their English language competency was ascertained to be of a sufficient level by the same criteria. Like overseas trained health professionals, these women had an excellent existing skill base and networks for referral should the need arise. However, in practice they did not have the time to interview as many women as originally anticipated due to heavy work and community commitments. Although they were willing to help and had good contacts for recruitment, the additional workload required for the research proved unfeasible in most cases.

### Conclusions on using bilingual workers

In summary, the advantage of working with bilingual workers was that they could help in participant recruitment, could provide the opportunity for participants to clarify questions, potentially allowing more comprehensive data to be collected [27], could work with the researchers about understanding the cultural meanings of questions and answers, and were able to give background information on the events in the local community. The bilingual/bicultural workers recruited participants through their own social networks of family, friends, and community members who met the selection criteria. Due to recruitment challenges with some ethnic minority communities, the temptation to use convenience samples that consist of community leaders, spokespersons and/or ethnic specific service providers is common. While it may be appealing to recruit a small number of carefully selected, easily accessed participants, concerns around selection bias must be acknowledged. Specific language proficiency or working for a community association does not necessarily mean that an individual represents a particular culture or is likely to reflect the majority of views in a community [13]. Although most people can easily identify a community to which they belong, agreeing on a spokesperson for that community, especially if they are to represent them in a community consultation or answer questions on their behalf, is much more difficult [36]. This is a particular concern for the most marginalised and isolated individuals, including refugees, who may be represented by a range of professionals such as lawyers, case workers, aid workers or interpreters that frequently speak on their behalf [33]. The opinions migrant and refugee women are often excluded when community spokespersons are used. It was important for WHFS to gather the views and priorities of newly arrived women who were either accessing the service or potentially could access the service. By working with bilingual/bicultural workers the project gained information from women that normally would not have participated in this type of research.

Working with the bilingual/bicultural workers to discuss and pilot the questionnaire was very beneficial. Words and phrases were more likely to have a shared meaning and be more easily understood by women from different cultures. Other researchers have also noted this [7,20]. Early in the study translating the questionnaire and consent forms was considered. However, as others have found [20], translations may not be an effective use of resources as participants can be illiterate in their own language and English or illiterate in their own language and literate in English. Working with bilingual/bicultural workers who read the questionnaire aloud overcame this difficulty.

We tried to ensure most of the bilingual/bicultural workers had a similar background to study participants, culturally or linguistically, as well as in the time they had been in Australia and their level of settlement. The matching of gender, ethnicity, and language is often desirable for cultural reasons, especially if sensitive topics such as alcohol and other drug use are under discussion and there is a strong possibility of social desirability bias [37]. Matching can include considerations of age, socio-economic status, status within the community, as well as matching of beliefs and views [1,8,9,12]. Although in many cases ethnic matching may be desirable, with sensitive material participants may not report or could under-report attitudes, beliefs and practices that are different to the norms and values of the interviewer, either for fear of offending the interviewer or other imagined repercussions that could arise from discussing stigmatised behaviour [1,2] Although we had tried to ensure similar backgrounds, it was difficult to ascertain the role/status of the bilingual/bicultural workers in their communities and how this may have influenced the results [22]. During debriefing sessions these issues were discussed as well as whether an interviewer unknown to the participants would be better. Some interviewers felt that participants may have been more honest with a stranger as there would be less pressure to give a socially acceptable response. However, when discussing social desirability bias more in depth with the workers, most of them felt that on the whole the women interviewed gave more honest answers with them than they would have done with a stranger. One explanation was that survey participants knew these women interviewers well and had discussed with them previously some of the issues highlighted in the questionnaire.

When working with more vulnerable groups, another difficulty in matching characteristics of participants with those of a bilingual/bicultural worker is that often the worker may be experiencing the same difficulties as the participants [20]. The worker may have little social support or time to conduct the interviews but may wish to take the job as it is extra money in to the household or for a variety of other reasons such as not wanting to offend the researcher [20]. This happened with two of the bilingual/bicultural workers. Researchers need to be aware that bilingual interviewers may say they are able to do this type of work because of cultural considerations around politeness and respecting those in perceived positions of power rather than because they have the time to undertake what is often quite difficult work.

An unforeseen benefit of using bilingual/bicultural workers was increasing the knowledge and experience of these women in raising issues about alcohol and other drug use, a much stigmatised topic in many ethnic communities. This knowledge and experience could be used both informally within their own communities and formally in their employment as these women were either planning or currently working in the health or social service sectors. This has also been found by other researchers [16].

Of surprise was that the majority of the interviews took place in English or a mixture of English and a community language. This is likely to be due to the arrival of a number of English speaking African community members at the time of the research. As the various English dialects spoken by these communities can be difficult to understand by Australian English speakers, interpreters or bilingual workers were essential in order to obtain information from these women. However, it has been noted by other researchers [16,17,19] that interviews often take place in two or even three languages with participants and interviewers switching between the languages. This may be an influence of acculturation, or that some concepts are more easily articulated in one language over another, or that English is the usual language of communication between the worker and the participant as they are in an English speaking country. The reasons for two or more languages being used in interviews and how that impacts on the quality of information gathered needs to be further explored.

The main disadvantage of using bilingual/bicultural workers was the time required to train, supervise and debrief them. For those workers who were new to the Australian employment system, arranging casual employment contracts and payment was time consuming. The interviews themselves were also time consuming to conduct and this has been noted by other researchers [21]. The time required is often not solely interview related as there is often an expectation of a general exchange of news, or to share beverages and/or food before or after the interview itself. While this can help build rapport, which ultimately provides more detailed information, this time needs to be taken into account as the number of planned interviews in a given time frame may be lower than originally anticipated. Table 4 summarises the advantages and disadvantages of using bilingual/bicultural workers and interpreters.

**Table 4:** Advantages and disadvantages of different types of bilingual workers used in the study

**Type of worker**	**Positives**	**Negatives**
**Professional Interpreters**	Verified fluency in English and community language	Expensive
	Well organised service for booking interpreters	No facility to help with participant recruitment
		Difficult to find interpreters for some languages
		Some problems with interpreters answering for participants
**Bilingual/Bicultural Students**	Provides practical experience for students in their field of study	Required intense supervision
	Often have good community networks to recruit interview participants	Limited professional experience on which to draw if client became distressed
	Often speak community languages where there is limited access to interpreters	Potential for social desirability bias as most women interviewed knew the student personally
		Level of language proficiency in community language was not accredited
**Overseas- trained Health Professionals**	Level of fluency in community language often recognised by an overseas university	Required some supervision
	Good understanding of confidentiality, boundaries & referral processes	Required intensive assistance with aspects of the Australian employment system
	Good professional experience on which to draw if participant became distressed	Potential for social desirability bias as most women surveyed knew them personally
	Often have experience in / interest in research, so already understand research protocols	
	Good community networks to find women to interview	
**Community- sector Bilingual Workers**	Good understanding of confidentiality, boundaries, referral processes	Heavy workload, so difficult to find time to interview new arrivals
	Good professional experience on which to draw if participant became distressed	Potential for social desirability bias as most women surveyed already knew them
	Good community networks	
	Require minimal supervision	

In reflecting on the elements of better practice in working with bilingual/bicultural workers outlined in Table 1, this study successfully incorporated most of these. The most difficult issue was limiting the number of bilingual interviewers in order to increase the dependability and credibility of the data gathered [19]. This is much easier to achieve in qualitative research where there are generally smaller numbers of participants. In this study 19 bilingual/bicultural workers were involved with the initial piloting and then administration of the questionnaire to 268 women. The large number of workers helped reduce selection bias and improve the heterogeneity of the snowball sample, as there was a broad range of snowball initiation points with limited links in each of the chains associated with the initiation point [33,38,39]. However, having so many interviewers, even to administer a questionnaire with few open questions, may have impacted on the quality of the data collected. The logistics of using bilingual/bicultural workers to collect data from larger numbers of participants does usually mean that more workers are needed in order to complete the project within a reasonable time frame. Other researchers have worked with more than one or two bilingual/bicultural interviewers [21,40], however there is little discussion in the literature on the logistics of administering questionnaires to larger multicultural groups who require bilingual/bicultural workers or interpreters in order to participate in research. This is an issue in many parts of Australia for health services that require quality information from all groups in a community, not just those with good English fluency. This is an area for future research.

Other impacts of using bilingual/bicultural workers in a project such as this would benefit from further research. One is gauging the refusal rate. The bilingual/bicultural workers had difficulty assessing how many potential participants refused to participate in the survey. For some potential participants it would socially unacceptable to give a direct refusal to the workers, so not showing up, making a reasonable excuse such as a sick child, or being busy were all reported as reasons for not being able to complete the survey. It was difficult to determine a refusal rate given these circumstances.

Another potential limitation was the impact of using bilingual/bicultural workers recruited from different sources. We did not detect any variations in the responses between different groups of bilingual/bicultural workers, for example whether all interviews done by one group yielded a particular result. However, we did not check this by reinterviewing participants using another category of bilingual workers, so this could be a short coming of the study which may have impacted on the quality of the data collected.

Not all the bilingual/bicultural workers surveyed women exclusively from their own ethnic language community, even though that had been the original expectation of the researchers. The workers had a much broader concept of community, incorporating other migrant women who they interacted with in places such as schools or churches. Given that almost 29 per cent of residents in the Perth metropolitan area have migrated from another country [41], it is to be expected that workers would know women from the wider migrant group, not just those from their own ethnic community. How this impacted on the data collected in the survey is unclear and could be further explored.

Recruitment difficulties and language issues both pose significant challenges to researchers working with culturally and linguistically diverse (CaLD) communities, and may even result in failure to include certain groups in research projects if these challenges prove insurmountable. This limits consideration of the individual and collective experiences of hard to reach groups. Substantial costs associated with preparing translated materials or employing professional interpreting services may also be incurred, imposing significant financial constraints on research budgets and further limiting the appeal of working with migrant groups [32]. Nevertheless, there is a clear need for health research with marginalised groups, so cost effective strategies need to be developed to maximise access and promote cross-cultural linkages between research teams and participants.

Although there is a significant body of literature describing the use of bilingual/bicultural workers in qualitative research [14,18], literature that discusses working with bilingual/bicultural workers for questionnaire administration in quantitative studies, especially for participants with no or minimal literacy, is a relatively new area. With that in mind, this paper can contribute to this area of research.

## Abbreviations

CaLD: Culturally and linguistically diverse; AOD: Alcohol and other drugs; WHFS: Women’s Health and Family Services; TIS: Translating and interpreting service; TAFE: Technical and further education.

## Competing interests

The authors declare that they have no competing interests.

## Authors’ contributions

SL conceived the study, participated in its design, co-ordination and analysis of data, and contributed to drafting the manuscript. ST participated in the design of the study and revision of the manuscript. CS-H drafted and revised the manuscript. All authors read and approved the final manuscript.

## Pre-publication history

The pre-publication history for this paper can be accessed here:

http://www.biomedcentral.com/1472-698X/14/11/prepub
